# Lack of Proinflammatory Cytokine Interleukin-6 or Tumor Necrosis Factor Receptor-1 Results in a Failure of the Innate Immune Response after Bacterial Meningitis

**DOI:** 10.1155/2016/7678542

**Published:** 2016-01-28

**Authors:** Lea-Jessica Albrecht, Simone C. Tauber, Julika Merres, Eugenia Kress, Matthias B. Stope, Sandra Jansen, Thomas Pufe, Lars-Ove Brandenburg

**Affiliations:** ^1^Department of Anatomy and Cell Biology, RWTH Aachen University, 52074 Aachen, Germany; ^2^Department of Neurology, RWTH University Hospital Aachen, 52074 Aachen, Germany; ^3^Department of Urology, University Medicine Greifswald, 17475 Greifswald, Germany

## Abstract

The most frequent pathogen that causes bacterial meningitis is the Gram-positive bacterium* Streptococcus pneumoniae*. By entering the brain, host cells will be activated and proinflammatory cytokines like interleukin-6 (IL-6) and tumor necrosis factor-*α* (TNF-*α*) are released. The goal of the current study was to examine the interaction between IL-6 and TNFR1 as receptor for TNF-*α* and the innate immune response in vivo in a model of* Streptococcus pneumoniae*-induced meningitis. For the experiments IL-6^−/−^, TNFR1^−/−^, and TNFR1-IL-6^−/−^ KO mice were used. Our results revealed higher mortality rates and bacterial burden after infection in TNFR1^−/−^, IL-6^−/−^, and TNFR1-IL-6^−/−^ mice and a decreased immune response including lower neutrophil infiltration in the meninges of TNFR1^−/−^ and TNFR1-IL-6^−/−^ mice in contrast to IL-6^−/−^ and wild type mice. Furthermore, the increased mortality of TNFR1^−/−^ and TNFR1-IL-6^−/−^ mice correlated with decreased glial cell activation compared to IL-6^−/−^ or wild type mice after pneumococcal meningitis. Altogether, the results show the importance of TNFR1 and IL-6 in the regulation of the innate immune response. The lack of TNFR1 and IL-6 results in higher mortality by weakened immune defence, whereas the lack of TNFR1 results in more severe impairment of the innate immune response than the lack of IL-6 alone.

## 1. Introduction

Bacterial meningitis is a serious infectious disease of the Central Nervous System (CNS). Despite the development of new antibiotics, bacterial meningitis is still burdened by a high mortality rate and frequent long-term sequelae such as cognitive deficits, hearing loss, or hydrocephalus malresorptivus [1]. The most frequent pathogen responsible for bacterial meningitis in adults with the highest rates of neurologic sequelae is the Gram-positive bacterium* Streptococcus pneumoniae *[2]. The pathophysiologic process in pneumococcal meningitis is complex with the immune response being a major key factor for both positive and negative effects on the outcome [3]. For the initiation of the inflammatory response and the detection of pathogens and their components, the cells of the innate immune system, glial cells, astrocytes, and microglia, play a very important role. Astrocytes and microglia are involved in the pathogen recognition and the activation of the innate immune response. They are the key regulators of the innate immune response within the brain because of their ability to release various factors supporting the host's immune defence and helping to coordinate the activation of different immune cells [4]. Astrocytes are furthermore required for structural support and the maintenance of the blood brain barrier (BBB). Microglia are considered to be CNS-resident macrophages and sensor cells that function as principal innate immune effector cells [5].

The major modulators for the regulation of the innate immune response including glial cell activation and immune cell recruitment are cytokines. By entering the brain, host cells will be activated and proinflammatory cytokines like interleukin-6 (IL-6) and tumor necrosis factor-*α* (TNF-*α*) are released. IL-6 is a multifunctional cytokine that is involved in regulation of immune response as well as acute-phase reaction and haematopoiesis [6]. The physiological function of IL-6 within the CNS is complex; IL-6 exerts neurotrophic and neuroprotective effects and yet can also function as a mediator of inflammation, demyelination, and astrogliosis, depending on the cellular context [7]. TNF-*α* is a central mediator of inflammation and tissue injury. It is involved in the pathogenesis of various acute and chronic inflammatory and neurodegenerative diseases including bacterial meningitis or Alzheimer's disease [8, 9]. Two different receptors mediate the functions of TNF-*α*. The tumor necrosis factor receptor-1 (TNFR1) induces the proinflammatory properties of TNF-*α*, while TNFR2 attenuates these properties [10]. Previous works have shown beneficial and detrimental effects of TNF-*α* in the host defence. It is essential for host defence against bacteria including* Streptococcus pneumoniae*, fungi, and parasites [11]. TNF-*α*-deficient mice showed increased mortality and stronger deficits in spatial memory possibly due to impaired neurogenesis after pneumococcal meningitis [12]. On the other hand, it has been suggested that TNF-*α* might be responsible for BBB dysfunction and decrease of cerebral blood flow in bacterial meningitis as well as neuronal cell death [13–15]. Nevertheless, our previous results showed a strong induction of cathelin-related antimicrobial peptide (CRAMP) expression after IL-6 and TNF-*α* treatment in glial cells [16]. Antimicrobial peptides as important part of the innate immune response fight against infiltrated pathogens. Furthermore they have immunomodulatory functions.

This study was designed to determine the role of TNFR1 and IL-6 in a mouse model of pneumococcal meningitis. We examined mortality, bacterial growth, and neutrophil granulocytes infiltration of infected IL-6^−/−^, TNFR1^−/−^, TNFR1-IL-6^−/−^, and wild type (WT) mice and characterized inflammatory responses by evaluating glial cell density, CRAMP, and pro- or anti-inflammatory cytokines expression.

## 2. Material and Methods

### 2.1. Animals

TNFR1^−/−^, IL-6^−/−^, TNFR1-IL-6^−/−^, or WT mice were bred in the Central Animal Care Facility of the RWTH Aachen University. The TNFR1^−/−^ and IL-6^−/−^-deficient mice have been originally presented by Rothe et al. [17] and Kopf et al. [18]. The mouse strains have a C57BL/6 background. The WT mice were backcrossed on the C57BL/6J background for at least five generations.

### 2.2. Mouse Model of Experimental Pneumococcal Meningitis [19]

TNFR1^−/−^, IL-6^−/−^, TNFR1-IL-6^−/−^, or WT mice (weight 19–23 g, aged 2-3 months) were anaesthetised with ketamine (100 mg/kg) and xylazine (20 mg/kg) and infected by injecting 10^4^ colony-forming units (CFU) of a* Streptococcus pneumoniae *D39 (type 2) strain in the subarachnoid space through the right frontolateral skull. Uninfected control animals were injected with 10 *μ*L of a sterile saline solution. All infected mice developed clinical signs of infection within 24 hours. The most sensitive sign of meningitis in mice was weight loss. There were no apparent behavioural abnormalities until ca. 18 h after infection, but mice gradually became lethargic between 18 and 24 h. Later than 24 h after infection, mice became severely lethargic and unable to walk and developed an opisthotonus. Epileptic seizures were observed but were infrequent. For the first set of experiments, the clinical state and survival time were closely monitored without antibiotic treatment. In a second set of experiments with the same infection regime, male TNFR1-, IL-6-, TNFR1-IL-6-deficient, or WT mice were infected and sacrificed 30 h after induction of meningitis without antibiotic treatment and were perfused either with 4% formalin for immunohistochemical analysis or with 0.9% NaCl solution for RNA isolation. Bacterial titers were evaluated 30 h after infection in tissue homogenates of cerebellum and spleen and in blood samples by plating 10-fold dilutions on blood agar plates and incubation for 24 h at 37°C and 5% CO_2_ (detection limit 10^2^ CFU/mL in tissue homogenates and 10^3^ CFU/mL in blood samples [8]). All animal experiments were approved by the Animal Care Committee of the University Hospital of Aachen and by the District Government in Recklinghausen, North Rhine-Westphalia, Germany.

### 2.3. Meningeal Inflammation Score

Meningeal inflammation was estimated by the invasion of granulocytes into the frontal interhemispheric region, the whole hippocampal fissure (both sides), 3 superficial meningeal regions over the convexities, and the third ventricle (complete distribution). One high power field (20 × objective) was scored in each region: no granulocytes: 0; <10 granulocytes: 1; 10 to 50 granulocytes: 2; and >50 granulocytes: 3. The scores of the individual regions were added (range of the score: 0–21) [19].

### 2.4. RNA Isolation and Real-Time RT-PCR and PCR Array

Total RNA was isolated using the peqGold Trifast reagent (Peqlab, Erlangen, Germany) according to the manufacturer's instructions. RNA samples were reverse-transcribed by Moloney murine leukemia virus (MMLV) reverse transcriptase (Fermentas, Burlington, Canada) and random hexamer primers (Invitrogen, Darmstadt, Germany). The cDNA products were used immediately for SYBR green (Applied Biosystems, Darmstadt, Germany) real-time RT-PCR. Gene expression was monitored using the StepOne Plus apparatus (Applied Biosystems, Darmstadt, Germany) according to manufacturer's protocol [20]. Relative quantification was performed using the ΔCt method which results in ratios between target genes and a housekeeping reference gene (TATA box binding protein for brain tissue (TBP)). The primers for glial fibrillary acid protein (GFAP), Integrin alpha M (Itgam), and CRAMP were manufactured by Qiagen (QT00101143, QT00156471, and QT00156571; QuantiTect Primer Assay; Qiagen, Hilden, Germany). The primers for TNF-*α*, TNFR2 (forward: 5′-GTCTTGACACCCTACAAACCGG-3′; reverse: 5′-ATTGGCCAGGAGGACACTTAGC-3′; annealing temperature: 59°C), interleukin-1*β* (IL-1*β*), IL-6, or interleukin-1 receptor antagonist (IL-1RA) were manufactured by Eurofins MWG Operon (Ebersberg, Germany; for primer sequences of TBP, TNF-*α*, IL-1*β*, and IL-6 see [21]; for IL-1RA see [19]). The specificity of the amplification reaction was determined by melting curve analysis.

### 2.5. Immunohistochemistry

Formalin-fixed and paraffin-embedded 5 *μ*m whole coronary brain sections were examined. For immunofluorescence staining, sections were deparaffinized, for Iba-1 staining pretreated for 10 minutes with microwaving in Tris/EDTA/Tween 20 buffer and after blocking with 5% normal goat serum in PBS incubated with either polyclonal rabbit anti-GFAP (1 : 9000; EnCor, Gainesville, FL, USA) or anti-Iba-1 (1 : 10.000; Wako, Neuss, Germany) overnight at 4°C. This was followed by incubation with the biotinylated secondary antibody (1 : 400; DAKO, Hamburg, Germany) and peroxidase-labeled streptavidin-biotin staining technique. For staining, Diaminobenzidine (DAB) was used. After counterstaining with haematoxylin, the slides were finally mounted with Aquatex (Boehringer, Mannheim, Germany).

### 2.6. Quantification of Immunoreactive Cells

The sections were examined blindly using a 10 × objective up to a 40 × objective. Only immunoreactive cells within the hippocampal formation were counted. An Analysis Software Imaging System (microscope Keyence BZ-9000; Keyence, Neu-Isenburg, Germany) was used to measure the area of the hippocampal formation. The densities of immunolabeled cells were expressed as the number of marked cells per square millimeter of the area measured. The density of labeled cells was evaluated in 4 coronal sections from each mouse.

### 2.7. Statistical Analysis

Survival time was expressed in hours and evaluated by generating a Kaplan-Meier plot that was statistically analyzed using the log-rank test. Bacterial titers were converted into log CFU/mL and compared by nonparametric *U* test. Immunohistochemical data were expressed as median and interquartile range. For statistical comparison, the nonparametric Mann-Whitney *U* test was used. The values for real-time RT-PCR analysis were expressed as the means of duplicate measurements to calculate means +/− SEM. For statistical comparison, ANOVA was performed followed by Bonferroni post hoc test. A value of *p* < 0.05 was considered statistically significant. For statistical calculation, GraphPad Prism 5.0 was used (Graph Pad Software, San Diego, CA, USA).

## 3. Results

### 3.1. TNFR1 and/or IL-6 Deficiency Resulted in Decreased Survival Time and Increased Bacterial Burden after Pneumococcal Meningitis

**Figure 1:**
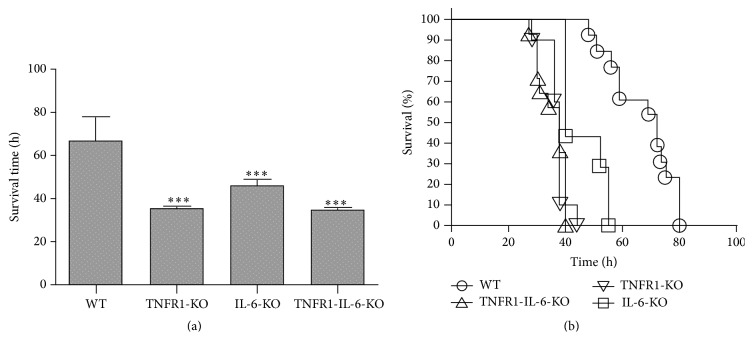
Mortality after pneumococcal meningitis. Wild type (WT), TNFR1-, IL-6, or TNFR1-IL-6 knockout (KO) mice were infected by injection of 10^4^ colony-forming units (CFU) of* Streptococcus pneumoniae* D39 (type 2) strain in the subarachnoid space. (a) Comparison of survival times revealed that WT mice lived significantly longer (WT, *n* = 16; TNFR1-KO, *n* = 14; IL-6-KO, *n* = 9; TNFR1-IL-6-KO, *n* = 18; median with percentile; Mann-Whitney *U* test; *p* < 0.0001 for TNFR1- or TNFR1-IL-6-KO versus WT and *p* = 0.0018 for IL-6-KO versus WT). (b) Mortality was significantly increased in TNFR1- and/or IL-6-KO animals (Kaplan-Meier-Curve; *p* < 0.0001 for TNFR1- and TNFR1-IL-6-KO, *p* = 0.0001 for IL-6-KO versus WT; log-rank test).

In a first set of experiments, survival time after pneumococcal meningitis was investigated. The survival time after infection was significantly shorter in TNFR1^−/−^, IL-6^−/−^, and TNFR1-IL-6^−/−^ (KO) mice compared to WT mice (36 (31/38), 40 (40/55), and 35 (30/40) versus 71 (57/75) h; medians (25th/75th percentiles); Mann-Whitney *U* test; *p* < 0.0001 for TNFR1^−/−^ and TNFR1-IL-6^−/−^ versus WT and *p* = 0.0018 for IL-6^−/−^ versus WT; Figure 1(a)). Fifty hours after infection, 92.3% of the WT animals were still alive, while all TNFR1^−/−^ and TNFR1-IL-6^−/−^ mice were dead and 28.6% of the IL-6^−/−^ mice were still alive (Figure 1(b)). The Kaplan-Meier curve revealed a significantly increased mortality in TNFR1^−/−^, IL-6^−/−^, and TNFR1-IL-6^−/−^ in comparison to WT mice (*p* < 0.0001 for TNFR1^−/−^ and TNFR1-IL-6^−/−^, *p* = 0.0001 for IL-6^−/−^ versus WT; log-rank test). In addition, we determined bacterial titers in homogenates of cerebellum and spleen and in blood samples from a separate set of TNFR1^−/−^, IL-6^−/−^, or TNFR1-IL-6^−/−^ or WT mice 30 h after infection. The results showed that TNFR1 and/or IL-6 deficiency resulted in a statistically significant higher bacterial burden in all investigated tissue compartments except in the cerebellum of TNFR1-IL-6^−/−^ mice compared to WT mice (Table 1).

### 3.2. Significant Decrease of Granulocyte Meningeal Invasion in TNFR1^−/−^ Mice after Pneumococcal Meningitis

Neutrophil granulocyte invasion was evaluated by evaluating CAE-staining 30 h after infection and transferring the results into a semiquantitative score reflecting meningeal inflammation (also see in Section 2): a strong granulocytic invasion of the meninges was observed in all infected mice 30 h after inoculation, whereas infiltration of granulocytes was absent in noninfected animals. There was a significant difference between meningeal inflammation of infected WT and TNFR1^−/−^ or TNFR1-IL-6^−/−^ mice: 12.4 ± 0.7 for WT, 7.3 ± 0.7 for TNFR1^−/−^ mice (*p* = 0.0003 compared to WT), 12.1 ± 0.9 for IL-6^−/−^-deficient mice, and 8.3 ± 0.8 (*p* = 0.0019; *n* = 10 each group; Mann-Whitney *U* test) reflecting a lower attraction of neutrophil granulocytes to the meninges in these mice in bacterial meningitis.

### 3.3. Decreased Glial Cell Activation during Pneumococcal Meningitis in TNFR1^−/−^ or TNFR1-IL-6^−/−^ Compared to WT Mice

**Figure 2:**
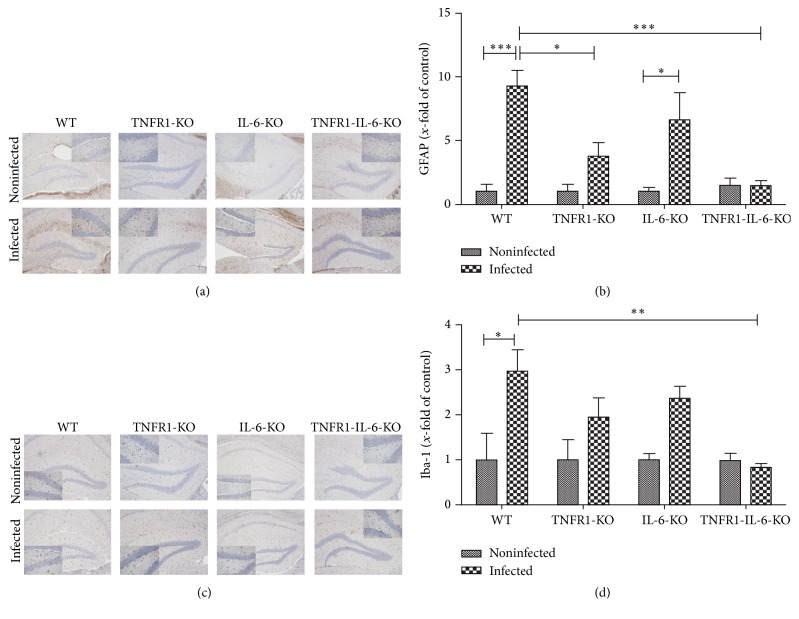
Glial cell density in the hippocampus 30 h after pneumococcal meningitis. Coronal brain sections stained with antibodies against GFAP to identify astrocytes (a) or Iba-1 to identify activated microglial cells (c) 30 h after infection with* Streptococcus pneumoniae.* A detailed section was included. GFAP (b) and Iba-1 (d) immunoreactive cells were quantified per mm^2^ area of the hippocampal formation of infected and healthy mice and represented as *x*-fold of control. Immunoreactive cells were normalized to noninfected controls. The asterisk indicates a significant difference between the animals groups (each group *n* = 5) mice as determined by ANOVA followed by Bonferroni test (^*∗*^ *p* < 0.05; ^*∗∗*^ *p* < 0.01; ^*∗∗∗*^ *p* < 0.001).

Thirty hours after infection, the degree of glial cell density was determined by immunohistochemistry of the astrocyte marker GFAP and the microglia marker ionized calcium binding adaptor molecule 1 (Iba-1) in the hippocampal formation as a clearly definable brain region especially involved in the pathogenesis of bacterial meningitis. As shown in Figures 2(a) and 2(c), the hippocampal formation of noninfected deficient as well as WT mice showed only very few GFAP or Iba-1 positive cells whereas the infection with* Streptococcus pneumoniae* resulted, as expected, in a strong increase in the number of GFAP or Iba-1-immunoreactive cells in WT mice. Quantification of astrocytes in the hippocampal formation revealed significantly less astrocytes in TNFR1^−/−^ or TNFR1-IL-6^−/−^ (KO) mice compared to the infected WT mice (9.3 ± 1.3-fold increase for infected WT, 3.8 ± 1.1-fold increase for TNFR1^−/−^, and 1.4 ± 0.4-fold increase for TNFR1-IL-6^−/−^ mice, *p* < 0.05 and *p* < 0.001; Figure 2(b)). The comparison between IL-6^−/−^ and WT mice did not reveal significant differences (6.6 ± 2.1-fold increase for IL-6^−/−^ mice). As expected, the number of microglial cells strongly increased in WT mice within the hippocampal formation 30 h after infection (2.9 ± 0.5-fold increase compared to noninfected WT, *p* < 0.05; Figure 2(d)). The three deficient mice strains showed a decrease of Iba-1 positive cells, but only TNFR1-IL-6^−/−^ mice reached a statistical significant difference compared to infected WT mice (0.8 ± 0.1-fold increase for infected TNFR1-IL-6^−/−^ mice, *p* < 0.01; Figure 2(d)). The level of the GFAP or Iba-1 quantification of the noninfected animals showed no significant differences (data not shown).

**Figure 3:**
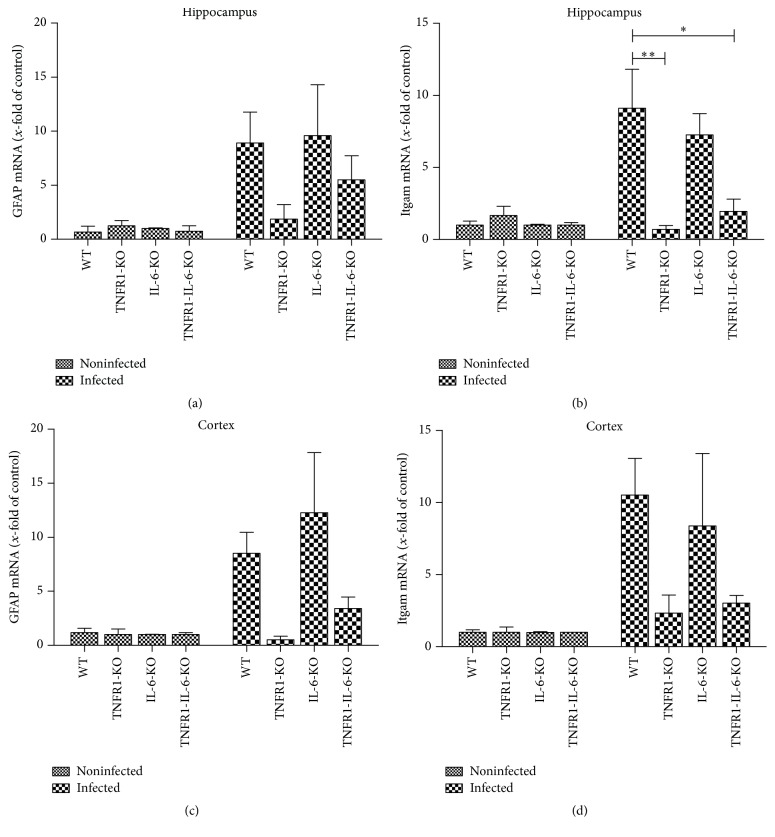
Gene expression of glial cell marker after pneumococcal meningitis. Analysis of astrocyte marker GFAP mRNA expression (a and c) and microglia marker Itgam (b and d) in the hippocampal formation and cortex of TNFR1-, IL-6-, or TNFR1-IL-6-KO and WT mice 30 h after induction of pneumococcal meningitis by real-time RT-PCR were assessed from five independent experiments in duplicate. The asterisk indicates a significant difference between infected WT and infected TNFR1- or TNFR1-IL-6-KO mice as determined by ANOVA followed by Bonferroni test (^*∗*^ *p* < 0.05; ^*∗∗*^ *p* < 0.01).

Furthermore, we analyzed the mRNA expression of GFAP as well as Integrin alpha M (Itgam or CD11b; [22]) as a marker for microglial cell activation in the hippocampal formation and cortex 30 h after infection. As shown in Figure 3, the pneumococcal infection resulted in a strong increase of GFAP and Itgam mRNA expression in the hippocampal formation and cortex of WT and IL-6^−/−^ mice compared to uninfected WT. The infected TNFR1^−/−^ and TNFR1-IL-6^−/−^ mice displayed a lower GFAP as well as Itgam mRNA expression in the hippocampus and cortex. The difference between infected WT and TNFR1^−/−^ or TNFR1-IL-6^−/−^ mice in the hippocampus was significant (9.1 ± 2.7-fold increase for infected WT, 0.7 ± 0.3-fold increase for infected TNFR1^−/−^, and 2 ± 0.9-fold increase for infected TNFR1-IL-6^−/−^ mice, *p* < 0.01 and *p* < 0.05; Figure 3(b)).

### 3.4. Reduced Innate Immune Response in TNFR1^−/−^ or TNFR1-IL-6^−/−^ Mice after Pneumococcal Meningitis

**Figure 4:**
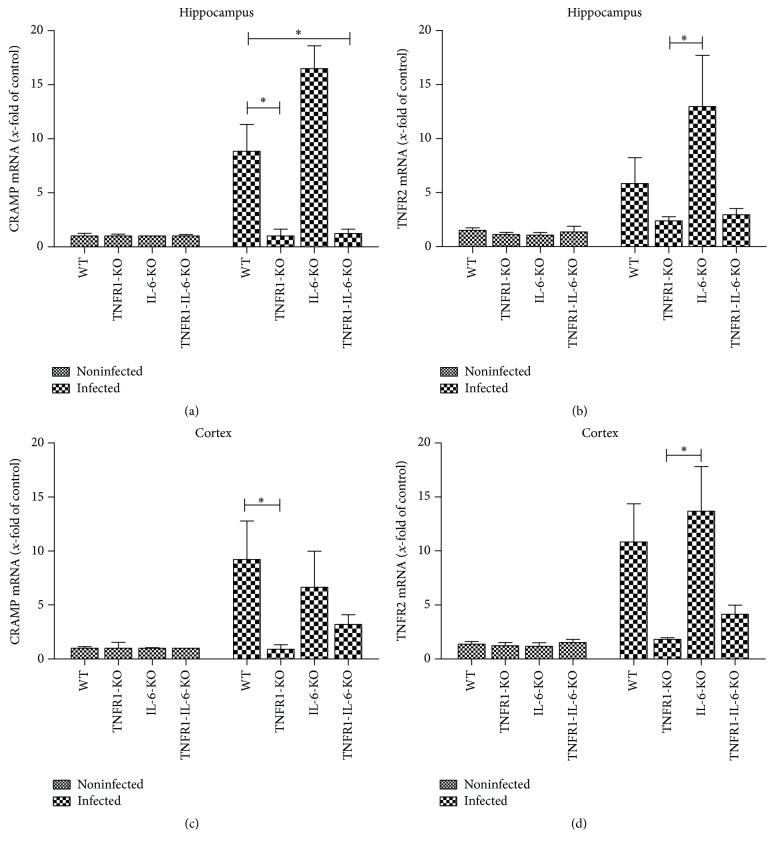
Expression of antimicrobial peptide CRAMP and TNFR2 after pneumococcal meningitis in hippocampus and cortex. 30 h after subarachnoid infection with* Streptococcus pneumoniae*, mRNA expression of CRAMP or TNFR2 was determined in the hippocampal formation (a and b) and cortex (c and d) from TNFR1-, IL-6-, or TNFR1-IL-6-KO and WT mice by real-time RT-PCR. Data were assessed from five independent experiments in duplicate. An asterisk indicates a significant difference between infected WT and infected TNFR1- or TNFR1-IL-6-KO mice as determined by ANOVA followed by Bonferroni test (^*∗*^ *p* < 0.05).

Furthermore, we analyzed the mRNA expression of an antimicrobial peptide as an important mediator of the innate immunity [23] in the hippocampus or cortex 30 h after pneumococcal infection. The antimicrobial peptide to be investigated was CRAMP [24]. As shown in Figures 4(a) and 4(c), the infection induced a strong increase of CRAMP mRNA expression in WT as well as IL-6^−/−^ mice in the hippocampal formation and cortex (8.8 ± 2- and 9.2 ± 3.5-fold induction for WT in the hippocampus and cortex; 16.5 ± 2.1- and 6.6 ± 3-fold induction for IL-6^−/−^ mice; Figures 4(a) and 4(c)). The difference between infected WT and TNFR1^−/−^ and TNFR1-IL-6^−/−^ mice in the hippocampus and infected WT and TNFR1^−/−^ mice in the cortex was significant (all *p* < 0.05; Figures 4(a) and 4(c)).

TNFR1 and TNFR2 mediate the activity of TNF-*α*. Therefore, we have determined the mRNA expression of TNFR2 in the hippocampal formation and cortex of TNFR1^−/−^, IL-6^−/−^, TNFR1-IL-6^−/−^, and WT mice after pneumococcal meningitis. As shown in Figures 4(b) and 4(d), pneumococcal infection resulted in an increase of TNFR2 mRNA expression in infected WT and IL-6^−/−^ mice, whereas the infected TNFR1^−/−^ and TNFR1-IL-6^−/−^ mice did not show an induction of the expression. The difference between infected IL-6^−/−^ and TNFR1^−/−^-deficient mice was significant (*p* < 0.05; Figures 4(b) and 4(d)).

**Figure 5:**
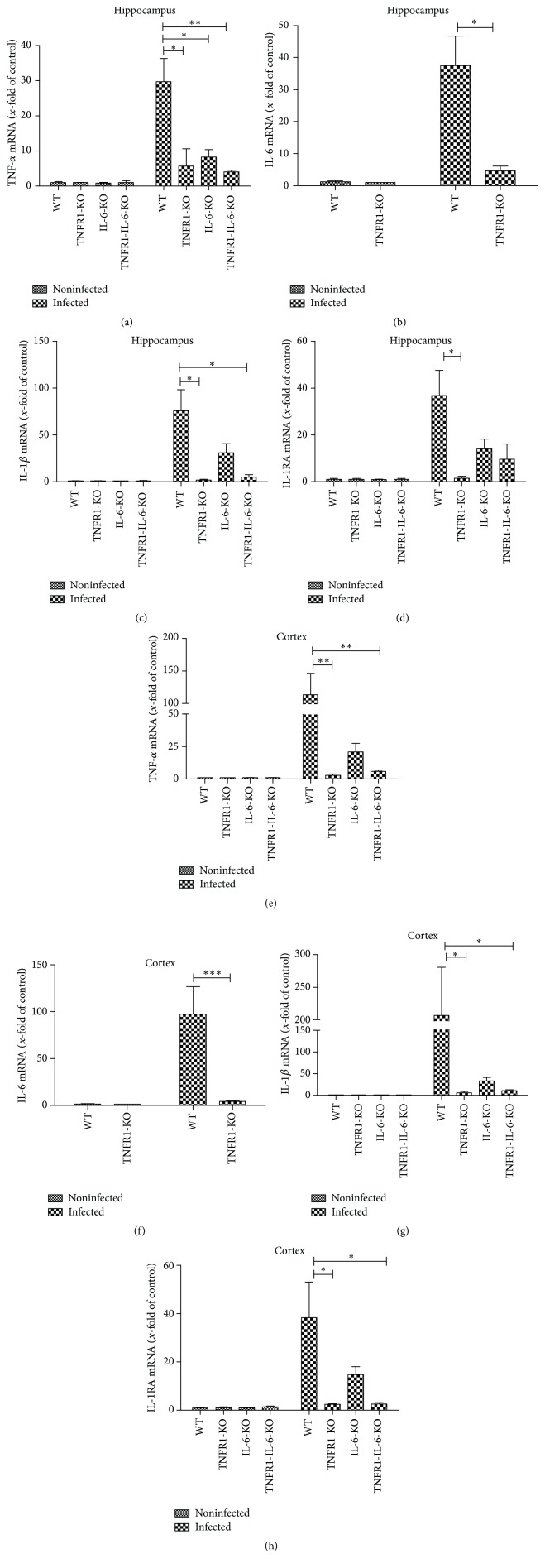
Analysis of innate immune response after pneumococcal meningitis in different brain regions. 30 h after subarachnoid infection with* Streptococcus pneumoniae*, mRNA expression of TNF-*α* (a and e), IL-6 (b and f), IL-1*β* (c and g), and IL-1RA (d and h) was determined in the hippocampal formation and cortex of TNFR1-, IL-6-, or TNFR1-IL-6-KO and WT mice by real-time RT-PCR. Data were assessed from five independent experiments in duplicate. An asterisk indicates a significant difference between infected WT and infected TNFR1-, IL-6-, or TNFR1-IL-6-KO mice as determined by ANOVA followed by Bonferroni test (^*∗*^ *p* < 0.05; ^*∗∗*^ *p* < 0.01; ^*∗∗∗*^ *p* < 0.001).

In a next set of experiments, we analyzed the mRNA expression of pro- and anti-inflammatory cytokines in the hippocampal formation and cortex of all four groups of mice after pneumococcal meningitis. At first, we examined the gene expression of the proinflammatory cytokines TNF-*α*, IL-6, and IL-1*β* as representatives of inflammatory mediators in pneumococcal meningitis. The infection resulted in a strong increase of the mRNA expression of TNF-*α* in the hippocampus and cortex of infected WT mice (29.7 ± 6.6-fold and 113 ± 33-fold induction of expression; Figures 5(a) and 5(e)). The infected TNFR1^−/−^, IL-6^−/−^, and TNFR1-IL-6^−/−^ mice showed a significant decrease of TNF-*α* mRNA expression compared to infected WT mice in the hippocampus and for TNFR1^−/−^ and TNFR1-IL-6^−/−^ mice in the cortex. As shown in Figures 5(b) and 5(f), the infection resulted in a strong increase of IL-6 mRNA expression in both the hippocampal formation and cortex of WT mice (37.5 ± 9- and 97.5 ± 29-fold induction for infected WT in the hippocampus and cortex) while the infected TNFR1^−/−^ mice showed a significant lower expression level (*p* < 0.05 and *p* < 0.001; Figures 5(b) and 5(f)). Similar results were obtained for the expression of IL-1*β*: meningitis resulted in a strong increase of IL-1*β* mRNA expression in the hippocampus and cortex in WT mice while the deficient mice strains displayed lower IL-1*β* levels with a significant difference for TNFR1^−/−^ and TNFR1-IL-6^−/−^ mice compared to infected WT mice (Figures 5(c) and 5(g)).

As a counterpart to the proinflammatory cytokines mentioned above, mRNA expression of the anti-inflammatory IL-1RA was carried out as it inhibits the proinflammatory response of IL-1*β* that is released primarily in response to exogenous agents [25]. As shown in Figures 5(d) and 5(h), the infection induced a strong increase of IL-1RA mRNA expression in the hippocampal formation and cortex of WT mice (35.8 ± 10- and 38.3 ± 14.7-fold induction for infected WT in the hippocampus and cortex) while the difference between WT and TNFR1^−/−^ in the hippocampus as well as WT and TNFR1^−/−^ and TNFR1-IL-6^−/−^ mice in the cortex reached statistical significance (all *p* < 0.05; Figures 5(d) and 5(h)).

## 4. Discussion

Bacterial meningitis is a life-threatening infection which is characterized by strong inflammation and neuronal damage with high mortality and long-term sequelae affecting up to 50% of survivors [1]. The entering of the subarachnoid space after crossing the BBB leads to bacterial proliferation and release of proinflammatory compounds by autolysis and secretion of mediators which enhance inflammation further and attract immune cells. Next to the direct toxic effects of the bacteria, the immune response also contributes to CNS injury [26]. The detection of bacteria or their components by the innate immune cells is mediated by so-called* pattern recognition receptors* (PRR) such as Toll-like receptors or formyl peptide receptors [5, 27, 28]. The activation of PRR results in induction and secretion of proinflammatory cytokines [29]. Cytokines such as IL-6 and TNF-*α* play an important role in bacterial meningitis and are synthesized in large quantities by monocytes, macrophages, and leukocytes in response to bacterial stimuli [30–32]. In the present study, the consequences of lack of TNFR1 and/or IL-6 were investigated in a well-characterized mouse model of pneumococcal meningitis [8, 19] and showed that the lack of both TNFR1 and IL-6 results in increased mortality and higher bacterial burden. TNF-*α* is secreted as a proinflammatory mediator, plays a crucial role in the initiation of the immune response, and strongly supports a faster pathogen elimination [33]. Both rodents and patients with bacterial meningitis show an increased amount of TNF-*α* in the cerebrospinal fluid (CSF) in the early phase of the disease [34] and TNF-*α*-deficient mice displayed an increased mortality after pneumococcal meningitis [12]. The proinflammatory properties of TNF-*α* are mediated by TNFR1 and TNFR2. The proinflammatory and pathogen-clearing activities of TNF-*α* are mainly mediated by activation of TNFR1, which is a strong activator of NF-kB, while TNFR2 may be more responsible for suppression of inflammation [35].

In this study, TNFR1 deficiency resulted in a higher mortality rate and increased bacterial load in meningitis compared to infected WT mice. In addition, the numbers of neutrophil granulocytes infiltrating the meninges were strongly reduced. In a model of experimental periodontitis, TNFR1 deficiency also resulted in a higher bacterial load, decreased proinflammatory cytokine expression such as IL-1*β*, and reduced polymorphonuclear leucocytes recruitment [36]. However, our results showed no increase of TNFR2 expression in TNFR1-deficient mice, whereas WT and IL-6-deficient mice displayed an increase of TNFR2 after infection. We interpreted this observation as a most likely compensative but in the end futile or at least insufficient upregulation in IL-6 animals since IL-6 displays pleiotropic effects both as a proinflammatory and anti-inflammatory modulator [6]. During the acute phase of meningitis, increased IL-6 levels can be found in CSF of patients [37]. Here, IL-6 deficiency led to increased mortality. Similarly, mortality was also increased in two other models of infection using either* Escherichia coli* or* Streptococcus pyogenes* infection in IL-6-deficient mice. In addition to the observed increased bacterial burden in IL-6^−/−^ mice with meningitis, the infection with* E. coli* also resulted in a higher accumulation of bacteria in the liver [38, 39]. However, the numbers of neutrophil granulocytes infiltrating the meninges were comparable between IL-6^−/−^ and WT mice in our work. Altogether, IL-6 is fundamentally required for the orchestration of the inflammatory process [40]. The lack of IL-6 should also result in a temporal and spatial disturbance of innate immune response. This is supported by our results, which show a higher mortality and bacterial load of the infected IL-6^−/−^ mice.

Several studies demonstrated the importance of glial cells in the development and regulation of inflammatory reactions in response to infections of the CNS [32]. Astrocytes belong to the well-characterized innate immune neuroglia that also liberate cytokines or chemokines [41]. Microglial cells as macrophages of the brain are being activated after the invasion of pathogens into the brain and also release a broad spectrum of cytokines and chemokines to activate other immune cells [22]. In bacterial meningitis, increased activation of astrocytes and microglia may aggravate neuronal cell death in the hippocampal formation by both induction of apoptosis and necrosis [26]. Contrarily, glial cells are important for recognizing and responding to microbial pathogens with subsequent phagocytosis or by secretion of neurotrophic factors to support neuroregeneration [32]. Our investigations revealed a reduced astroglial and microglial activation in the hippocampus of TNFR1^−/−^ and TNFR1-IL-6^−/−^ mice after pneumococcal meningitis, whereas the infected IL-6^−/−^ and WT mice showed no significant difference in glial cell activation. This observation confirms the importance of the TNFR1 for the glial cell activation. In a mouse model of LPS-sensitized hypoxic-ischemic injury in the immature brain TNFR1-deficient mice also displayed reduced cell densities of astrocytes and microglia whereas TNFR2-deficient mice did not differ from WT mice [42]. Another study also revealed an inhibition of microglial activation expression and cerebral monocytes recruitment after periphery organ inflammation in TNFR1-deficient mice, whereas no inhibition was observed in TNFR2-deficient mice [43]. The activation of TNFR2 rather promoted the induction of anti-inflammatory pathways in microglial cells [44].

Analysis of the innate immune response showed in TNFR1^−/−^ and TNFR1-IL-6^−/−^ mice an impaired expression of both proinflammatory and anti-inflammatory markers. The general immune cascade seems to be strongly weakened. The activation of TNFR1 and the signal transduction leads usually to activation of NF-kB and AP-1 and further to activation of proinflammatory and antiapoptotic genes [6, 10]. So, TNFR1 deficiency most likely is the reason for the only mild increase of cytokine expression after pneumococcal meningitis leading to the impaired immune response. However, pharmacological downregulation of TNF-*α* levels in the CSF decreased mortality and neuronal damage and improved the learning performance 3 weeks after infection in a rat model of pneumococcal meningitis [45] providing evidence for better outcome by reducing TNF-*α* levels. This underlines the thin line between beneficial and detrimental effects of TNF-*α*. In contrast to lowering the TNF-*α* levels, the complete absence of TNF-*α* or its receptor TNFR1 led to detrimental effects. This included also the absence of IL-1*β* and IL-1RA expression. For IL-1RA, a recent work of Barichello et al. showed that interleukin-1*β* receptor antagonism prevents cognitive impairment following experimental bacterial meningitis [46]. Furthermore, TNFR1^−/−^ and TNFR1-IL-6^−/−^ mice showed only a weak increase of the antimicrobial peptide CRAMP after infection while in contrast IL-6^−/−^ mice displayed CRAMP levels similar to the ones of WT mice. The antimicrobial peptides are an important part of the innate immune system with direct antimicrobial activity but also immunomodulatory properties [23]. Our previous studies showed an increased expression of the proinflammatory cytokines TNF-*α* and IL-6 in CRAMP-deficient mice after pneumococcal meningitis [8]. In addition, the expression of CRAMP was induced by TNF-*α* and IL-6 treatment in glial and meningeal cells [16, 47], whereas vice versa CRAMP stimulation also induced the expression of both cytokines [48] providing evidence of a direct relationship between CRAMP and these cytokines. While the higher bacterial burden in TNFR1-deficient mice might be the result of low levels of CRAMP and thereby lower support in antimicrobial activity, IL-6^−/−^ mice with CRAMP levels similar to WT animals also displayed increased bacterial titers. Either CRAMP activity was impaired under these circumstances or the higher bacterial burden might be the result of the lower grade of infiltration of meningeal neutrophil granulocytes.

Taken together, the present study provides evidence for the important role of TNFR1 and IL-6 as part of the innate immune response after bacterial infection. The lack of TNFR1 leads to a collapse of robust parts of the immune response and caused as a consequence a worse outcome due to a higher mortality. Although the absence of IL-6 does not seem to cause as severe modulations as TNFR1 deficiency, it still impaired the immune defence strongly enough to also result in a higher mortality rate. The compensatory mechanisms in IL-6^−/−^ mice like, for example, upregulation of TNFR2 appear to be futile, so that in the end factors like the increased bacterial load lead to an earlier decrease than in WT mice. The observations in double deficient TNRF1/IL-6^−/−^ mice resemble the ones from the TNFR1^−/−^ alone hinting towards a predominance of TNRF1 in comparison to IL-6 alone. Our results provide interesting insights into the function of the innate immune system during the course of bacterial meningitis.

## Figures and Tables

**Table 1:** Bacterial titers after pneumococcal meningitis.

Bacterial titer (log CFU/mL)			
	Blood	Spleen	Cerebellum
WT	5.4 (4.3/6.75)	5.5 (4.88/6.23)	6 (5.3/6.48)
TNFR1-KO	9.65 (8.35/10.23)^*∗∗∗*^	9.65 (8.2/10.23)^*∗∗∗*^	7.8 (7/9.75)^*∗∗∗*^
IL-6-KO	10.5 (6.74/11.05)^*∗*^	8.7 (7/10.21)^*∗*^	8.74 (7/10.64)^*∗*^
TNFR1-IL-6-KO	7.65 (6.25/8.25)^*∗∗*^	7.7 (7/8.3)^*∗*^	8.3 (6/10)

Data are presented as median (25th/75th percentile). Please note significantly increased bacterial titers in infected TNFR1-, IL-6-, or TNR1-IL-6-KO compared to infected WT mice (*n* = 10 each group; ^*∗*^ *p* < 0.05; ^*∗∗*^ *p* < 0.01 for cerebellum; ^*∗∗∗*^ *p* < 0.001; Mann-Whitney *U* test) (CFU, colony-forming units; WT, wild type; and KO, knockout).

## Abbreviations

CRAMP: Cathelin-related antimicrobial peptide
GFAP: Glial fibrillary acidic protein
Iba-1: Ionized calcium binding adaptor molecule 1
IL-6: Interleukin-6
Itgam: Integrin alpha M
KO: Knockout
WT: Wild type
PRR: Pattern recognition receptors
TNFR1: Tumor necrosis factor receptor-1.
